# *Sox* Genes Show Spatiotemporal Expression during Murine Tongue and Eyelid Development

**DOI:** 10.1155/2018/1601363

**Published:** 2018-10-09

**Authors:** Ryuichi Ishikawa, Maiko Kawasaki, Katsushige Kawasaki, Akane Yamada, Supaluk Trakanant, Fumiya Meguro, Atsushi Kitamura, Takehisa Kudo, Takeyasu Maeda, Atsushi Ohazama

**Affiliations:** ^1^Division of Oral Anatomy, Department of Oral Life Science, Niigata University Graduate School of Medical and Dental Sciences, Niigata, Japan; ^2^Research Center for Advanced Oral Science, Department of Oral Life Science, Niigata University Graduate School of Medical and Dental Sciences, Niigata, Japan

## Abstract

The tongue is a critical organ, involved in functions such as speaking, swallowing, mastication, and degustation. Although *Sox* genes are known to play critical roles in many biological processes, including organogenesis, the expression of the *Sox* family members during tongue development remains unclear. We therefore performed a comparative *in situ* hybridization analysis of 17 *Sox* genes (*Sox1–14, 17, 18,* and *21*) during murine tongue development. *Sox2, 4, 6, 8, 9, 10, 11, 12,* and *21* were found to be expressed in the tongue epithelium, whereas *Sox2, 4–6, 8–11, 13,* and *21* showed expression in the mesenchyme of the developing tongue. Expression of *Sox1, 4, 6, 8*–*12*, and *21* were observed in the developing tongue muscle. *Sox5* and *13* showed expression only at E12, while *Sox1* expression was observed only on E18. *Sox6, 8, 9*, and *12* showed expression at several stages. Although the expression of *Sox2*, *4*, *10*, *11*, and *21* was detected during all the four stages of tongue development, their expression patterns differed among the stages. We thus identified a dynamic spatiotemporal expression pattern of the *Sox* genes during murine tongue development. To understand whether *Sox* genes are involved in the development of other craniofacial organs through similar roles to those in tongue development, we also examined the expression of *Sox* genes in eyelid primordia, which also contain epithelium, mesenchyme, and muscle. However, expression patterns and timing of *Sox* genes differed between tongue and eyelid development. *Sox* genes are thus related to organogenesis through different functions in each craniofacial organ.

## 1. Introduction

The tongue plays a critical role in speaking, swallowing, mastication, and degustation. Malformations of the tongue, including macroglossia, hypoglossia, and aglossia, are seen as congenital defects. Therefore, it is crucial to understand the molecular mechanisms involved in tongue development; however, these mechanisms remain unclear.

The mammalian tongue is composed of the epithelium, connective tissue, and striated muscle. The connective tissue and vasculature of the tongue are derived from the cranial neural crest, whereas most tongue muscles are formed by myoblasts that migrate from the occipital somites [1]. It is believed that there is an interaction between neural crest-derived and myogenic cells during tongue development [2, 3].

In mice, tongue development begins at embryonic day (E) 10.5–11.0 with the formation of medial lingual swelling from the first branchial arch. Next, lateral lingual swellings are formed on each side of the median tongue bud. They overgrow the medial lingual swelling and eventually fuse to form the anterior two-thirds of the tongue. The medial lingual swelling does not form any identifiable part of the adult tongue. The third branchial arch gives rise to the copula and the hypopharyngeal eminence. Subsequently, the hypopharyngeal eminence overgrows the copula, which in turn disappears progressively. Consequently, the rostral part of the hypopharyngeal eminence develops into the posterior third of the tongue [2, 3, 4]. Thus, the embryonic origin in the anterior two-thirds and posterior third of the tongue differs. In addition, the anterior two-thirds of the tongue is mobile, whereas the posterior third is relatively immobile.

Tongue muscles are classified as either intrinsic or extrinsic. They are bilateral and separated by the lingual septum, which consists of fibrous connective tissue. Four different types of tongue papillae can be distinguished: fungiform, circumvallate, foliate, and filiform. They are known to develop through epithelial-mesenchymal interactions. Although the lingual epithelium is histologically homogeneous during the early stages of tongue development, the papillae become distinct and protrude from the dorsum of the tongue on approximately E12. A single circumvallate papilla is located at the center of the terminal sulcus. Fungiform papillae are present on the anterior part of the tongue in a pattern of longitudinal rows bracketing the median furrow. Unlike the other three types of papillae, filiform papillae cover the entire dorsal surface of the tongue.

Sox proteins are characterized by a highly conserved DNA-binding motif called high-mobility group domain. To date, 20 *Sox* genes have been identified in mice. Members of the *Sox* gene family show dynamic and diverse expression patterns during development, and they play multiple roles during development, as evidenced by mutation analyses in mice [5, 6]. Spatiotemporal expression patterns of *Sox* genes have been reported in tooth and pancreas development [7, 8]. In the trunk and limb region, it has been reported that *Sox6* is involved in skeletal muscle formation, while *Sox7, 15, 17,* and *18* have been shown to be related to function of satellite cells [9–13]. Although *Sox2* has been reported to be involved in the development of tongue papillae [14], the expression pattern of other *Sox* genes during the entire development of the tongue remains unclear. Therefore, we performed a comparative *in situ* hybridization analysis of 17 *Sox* genes (*Sox1–14, 17, 18,* and *21*) during murine tongue development and identified their dynamic spatiotemporal expression.

## 2. Materials and Methods

### 2.1. Production and Analysis of Mice

CD-1 strain mice were used in this study. The noon time of the day on which the plugs were detected was considered as E0.5. To accurately assess the age of the embryos, somite pairs were counted, and the stage was confirmed using morphological criteria, e.g., relative sizes of maxillary and mandibular primordia, extent of nasal placode invagination, and size of limb buds.

### 2.2. *In Situ* Hybridization

Embryo heads were fixed in 4% buffered paraformaldehyde, wax embedded, and serially sectioned at 7 *µ*m. Decalcification using 0.5 M EDTA (ethylenediaminetetraacetic acid, pH 7.6) was performed after fixation of the newborn mice. Sections were split over three to ten slides and prepared for histology or radioactive *in situ* hybridization. *In situ* hybridization with [^35^S]UTP-labeled riboprobes (Table 1) was performed as previously described [15]. Briefly, the slides were pretreated with proteinase K and acetic anhydride to reduce the background. Hybridization was carried out overnight in a humidified chamber at 55°C. The slides were then washed and treated with RNAse A for 30 min at 37°C to remove any nonspecifically bound probe. The high stringency washes were repeated, and the sections were then dehydrated. The slides were air-dried and dipped in Ilford K-5 photographic emulsion. Autoradiography was performed by exposing the sections in a light-tight box at 4°C for 10–14 days. Slides were developed using Kodak D19, fixed in Kodak Unifix, and counterstained with malachite green or hematoxylin.

## 3. Results

The expression of *Sox3* and *14* was not detected in the developing tongue (data not shown).

### 3.1. E11

**Figure 1:**
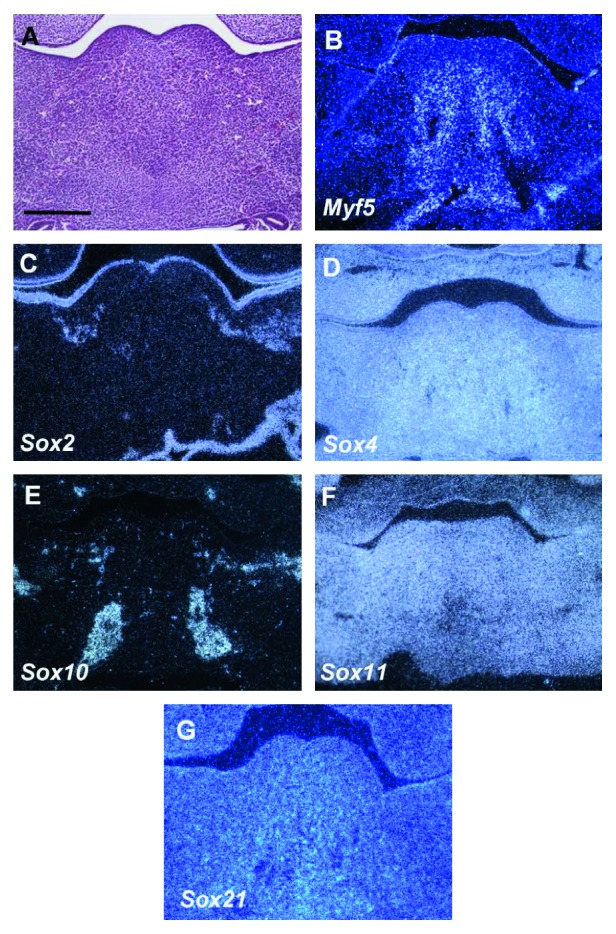
Expression of *Sox* genes in tongue development at E11. Frontal sections showing histology (a) and *in situ* hybridization (b–g) in wild-type tongue at E11. Scale bar: 300 *μ*m for (a–g).

The tongue primordium is firstly recognizable on the mandibular processes as a small protrusion (Figure 1). To identify muscle progenitor cells, *Myf5* expression was examined in the developing tongue. Two domains of *Myf5* expression were observed in the center of the tongue primordium, which was found to be further divided into four domains in the caudal region (Figure 1). *Sox4, 11,* and *21* were ubiquitously expressed in the developing tongue (Figures 1, 1, and 1). *Sox2* and *10* expressions were observed in the mesenchyme at the lateral side of the tongue primordium, whereas *Sox2* also showed the expression in the epithelium (Figures 1 and 1). *Sox1, 5, 6, 8, 9,* and *12–14* showed no expression in the developing tongue at E11 (data not shown).

Since distinct morphological structures are present in the developing tongue along the anterior-posterior axis, it is likely that the expression patterns differ among the various regions of the developing tongue. Therefore, we examined the expression patterns of the *Sox* gene family members in the anterior, middle, and posterior regions of the tongue.

### 3.2. E12

**Figure 2:**
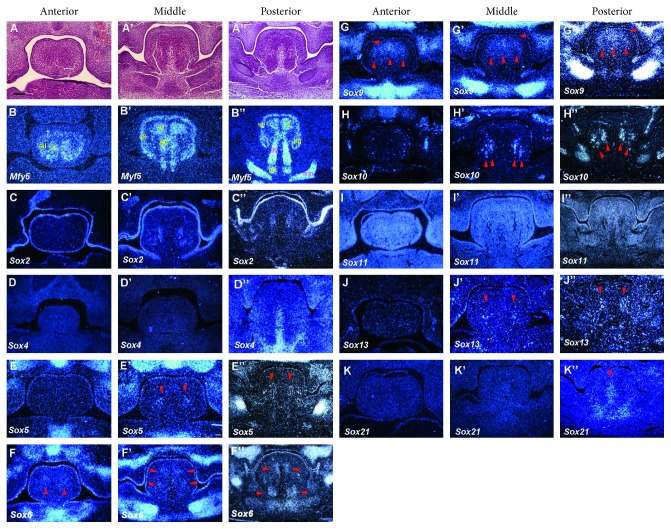
Expression of Sox genes in tongue development at E12. Frontal sections showing histology ((a)–(a″)) and *in situ* hybridization ((b)–(k″)) at the anterior, middle, and posterior regions of mandible in wild-type at E12. Vertical and transverse muscle (vt), genioglossus muscle (gg), superior and inferior longitudinal muscle (sil), geniohyoid muscle (gh), mylohyoid (mm), and hyoglossus muscle (hg). Arrowheads indicate weak expression. Scale bar: 125 *μ*m for (a)–(k″).

At E12, condensation of mesenchymal cells was observed in the spatulate tongue (Figures 2–2). A primitive muscle was identified only in the middle and posterior regions of the developing tongue (Figures 2–2). *Myf5* was expressed in primitive vertical and transverse (vt), genioglossus (gg), hyoglossus (hg), superior and inferior longitudinal (sil), geniohyoid (gh), and mylohyoid (mm) muscle regions (Figures 2–2). *Sox2* was expressed in the mesenchyme between the sil and gg muscles in the middle and posterior regions of the tongue as well as in the epithelium throughout the tongue (Figures 2–2). The expression of *Sox4* was found in the septum of the posterior region of the tongue (Figures 2–2). *Sox5* was weakly expressed in the mesenchyme between the sil and gg muscles in the middle and posterior regions of the tongue (Figures 2–2). Weak expression of *Sox6* was observed in the epithelium and vt and gg muscles (Figures 2–2). *Sox9* expression was observed in vt muscles, mesenchyme between vt muscles, and epithelium of the entire tongue (Figures 2–2). *Sox10* expression was detected in the mesenchyme between the sil and gg muscles in the middle and posterior regions of the tongue as well as in the hg muscle region (Figures 2–2). *Sox11* was ubiquitously expressed in the developing tongue (Figures 2–2). Weak expression of *Sox13* was observed in the mesenchyme between the sil and gg muscles in the middle and posterior regions of the tongue (Figures 2–2). *Sox21* was expressed in the lingual septum of the posterior developing tongue (Figures 2–2). *Sox1, 8,* and *12* showed no expression in the developing tongue at E12 (data not shown). *Sox5, 6,* and *9* also showed strong expressions in the presumptive Meckel's cartilage region.

### 3.3. E13

**Figure 3:**
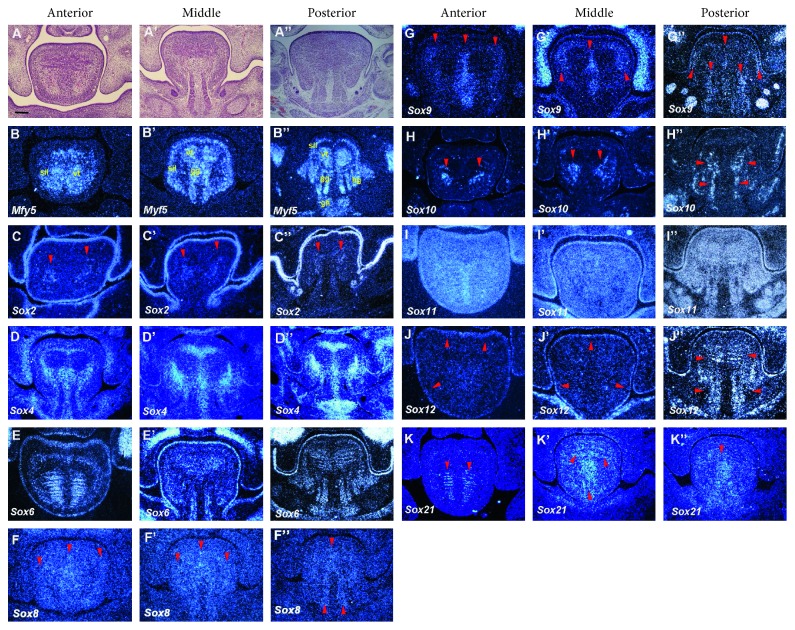
Expression of *Sox* genes in tongue development at E13. Frontal sections showing histology ((a)–(a″)) and *in situ* hybridization ((b)–(k″)) at the anterior, middle and posterior regions of mandible in wild-type at E13. Vertical and transverse muscle (vt), genioglossus muscle (gg), superior and inferior longitudinal muscle (sil), geniohyoid muscle (gh), hyoglossus muscle (hg). Arrowheads indicate weak expression. Scale bar: 125 *μ*m for (a)–(k″).

The vt, gg, and hg muscles were histologically identifiable at E13 (Figures 3(a)–3). *Myf5* expression in the anterior and middle regions of the tongue was comparable to that at E12, while it was divided into two expression domains in the gg muscles (Figures 3–3). *Sox2* was expressed in the mesenchyme between the vt and sil muscles and epithelium of the entire tongue (Figures 3–3). The expression of *Sox4* was observed in the mesenchyme surrounding the vt muscles, including the lingual septum throughout the tongue (Figures 3–3). *Sox6* was expressed in the epithelium and the vt, gg, hg, and sil muscles of the entire developing tongue (Figures 3–3). *Sox8* was weakly expressed in the septum of the entire tongue, the sil muscle in the anterior and middle tongue, and the gg muscle in the posterior regions of the tongue (Figures 3–3). Expression of *Sox9* was detected in the lingual septum in the entire tongue, while it was also weakly expressed in the sil muscle of the entire tongue (Figures 3–3. Weak expression of *Sox9* was also found in the gg muscle region of the posterior tongue (Figures 3). *Sox10* was expressed in the mesenchyme between the vt and sil muscles in the entire tongue, as well as in the gg muscle in the posterior region of the tongue (Figures 3–3). *Sox11* showed ubiquitous expression in the entire developing tongue (Figures 3–3). *Sox12* was expressed in the epithelium of the entire tongue, and the vt and gg muscles in the posterior region of the tongue (Figures 3–3). *Sox21* expression was observed in the vt muscle in the anterior and middle tongue, while it was found in the septum in the middle and posterior tongue (Figures 3–3). *Sox1, 5,* and *13* showed no expression in the developing tongue at E13 (data not shown).

### 3.4. E14

**Figure 4:**
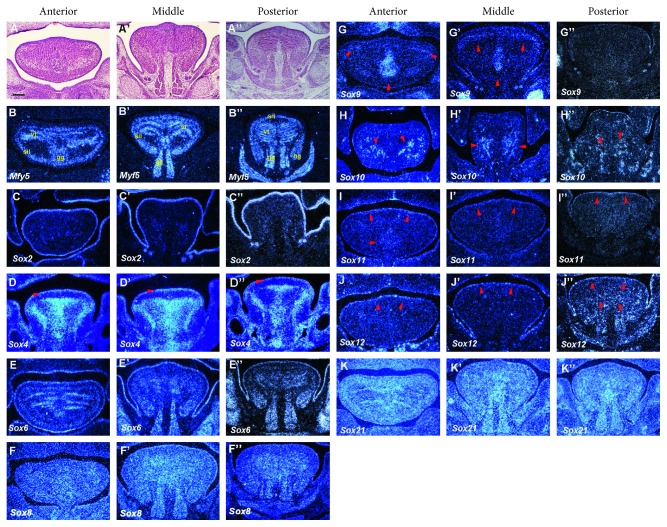
Expression of *Sox* genes in tongue development at E14. Frontal sections showing histology ((a)–(a″)) and *in situ* hybridization ((b)–(k″)) at the anterior, middle and posterior regions of mandible in wild-type at E14. Vertical and transverse muscle (vt), genioglossus muscle (gg), superior and inferior longitudinal muscle (sil), geniohyoid muscle (gh), hyoglossus muscle (hg). Arrowheads indicate weak expression. Scale bar: 300 *μ*m for (a)–(k″).

Each muscle was found to be more morphologically obvious at E14 (Figures 4-4). *Myf5* was also expressed in the gg muscle in the anterior tongue (Figures 4). *Myf5* expression was divided into several domains in the vt muscle region of the entire developing tongue (Figures 4 and 4). *Sox2* showed expression in the epithelium of the entire tongue (Figures 4–4). Expression of *Sox4* was observed in the mesenchyme surrounding the vt muscles, including the septum throughout the tongue, as well as in the epithelium (Figures 4–4). *Sox6* showed expression in the vt, hg, and gg muscles in the entire tongue, as well as in the epithelium (Figures 4–4). *Sox8* showed weak and ubiquitous expression in the entire tongue (Figures 4–4). In the anterior and middle regions of the tongue, *Sox9* was strongly expressed in the gg muscles, and weakly expressed in the sil muscle (Figures 4–4). Expression of *Sox10* was observed in the mesenchyme underneath the vt muscles in the anterior and middle regions of the tongue, and in the gg muscle in the posterior region (Figures 4–4). Weak expression of *Sox11* was detected in the epithelium of the entire tongue and gg muscles in the anterior region of the tongue (Figures 4–4). *Sox12* showed weak expression in the epithelium of the entire tongue and the gg muscles in the posterior part of the tongue (Figures 4–4). *Sox21* showed ubiquitous expression in the entire developing tongue (Figures 4–4). *Sox1, 5,* and *13* showed no expression in the developing tongue at E14 (data not shown).

### 3.5. E18

**Figure 5:**
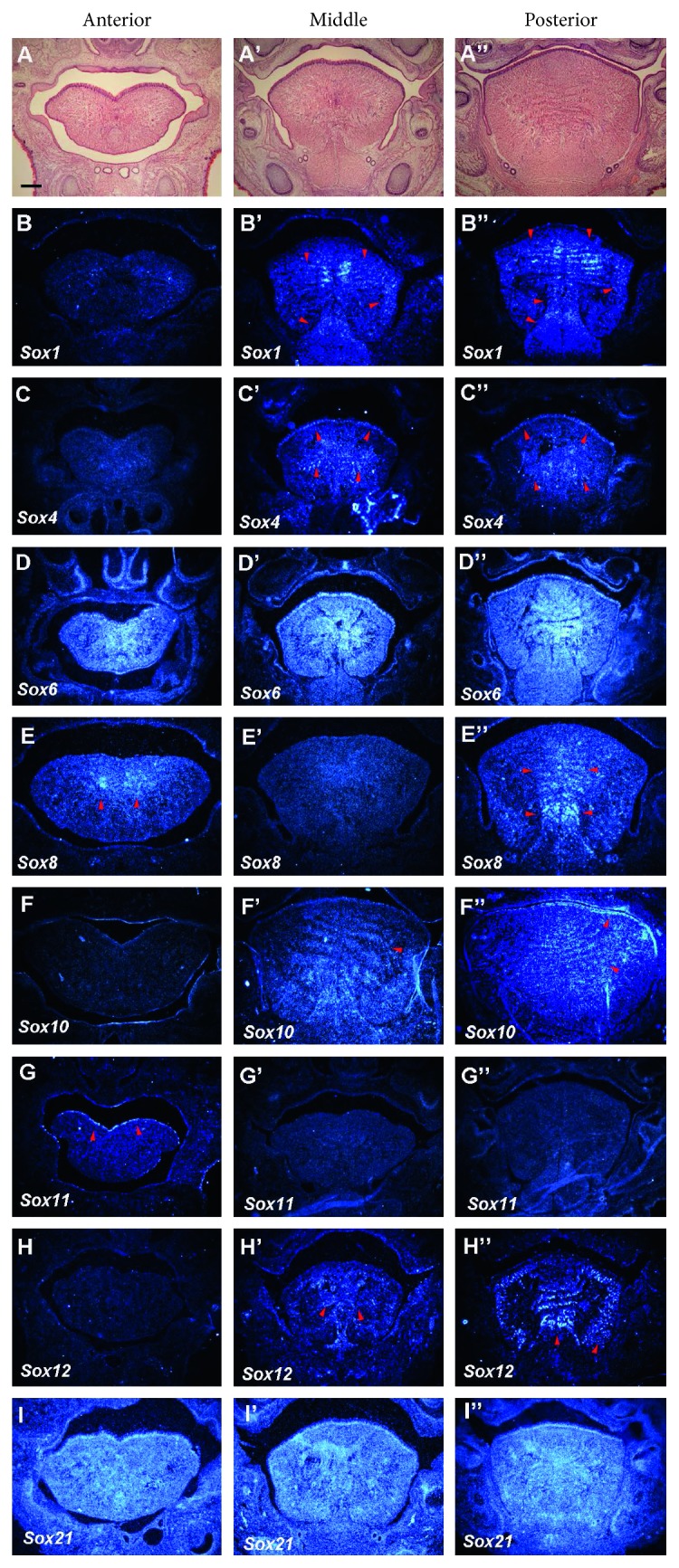
Expression of *Sox* genes in tongue development at E18. Frontal sections showing histology ((a)–(a″)) and *in situ* hybridization ((b)–(i″)) at the anterior, middle, and posterior regions of mandible in wild-type at E18. Arrowheads indicate weak expression. Scale bar: 700 *μ*m for (a)–(i″).

Most tongue muscle progenitor cells had already been differentiated into myoblasts by E18. Therefore, only weak expression of *Myf5* could be observed in the tongue at E18 (data not shown). *Sox1* showed weak expression in the sil, vt, gg, and gh muscles in the middle and posterior regions of the tongue (Figures 5–5). *Sox2* has been shown to be expressed in the epithelium at this developmental stage [14]. *Sox4* showed weak expression in the epithelium and vt muscles in the middle and posterior regions of the tongue (Figures 5–5). *Sox6* showed ubiquitous expression in the entire tongue (Figures 5–5). Expression of *Sox8* was detected in the mesial part of the vt muscles in the anterior region of the tongue, as well as in both the mesial part of the vt muscles and the rostral part of the gg muscle in the posterior region of the tongue (Figures 5–5). Weak expression of *Sox10* was found in the vt muscles in the middle and posterior regions of the tongue, as well as in the epithelium in the posterior region of the tongue (Figures 5–5). *Sox11* was weakly expressed in the dorsum epithelium in the anterior region of the tongue (Figures 5–5). *Sox12* expression was detected in the vt muscles in the middle region of the tongue, as well as in the vt and sil muscles in the posterior region of the tongue (Figures 5–5). *Sox21* showed ubiquitous expression in the entire developing tongue (Figures 5–5). *Sox2, 5, 9,* and *13* showed no expression in the developing tongue at E18 (data not shown).

*Sox7, 17,* and *18* belong to group F of the *Sox* gene family (SoxF). Members of SoxF have been shown to regulate blood and lymphatic vascular development [16–18]. A punctate expression pattern of *Sox7, 17,* and *18* was seen throughout the developing tongue, and it probably represents vascularization of the tissue (data not shown).

### 3.6. *Sox* Genes in Eyelid Development

**Figure 6:**
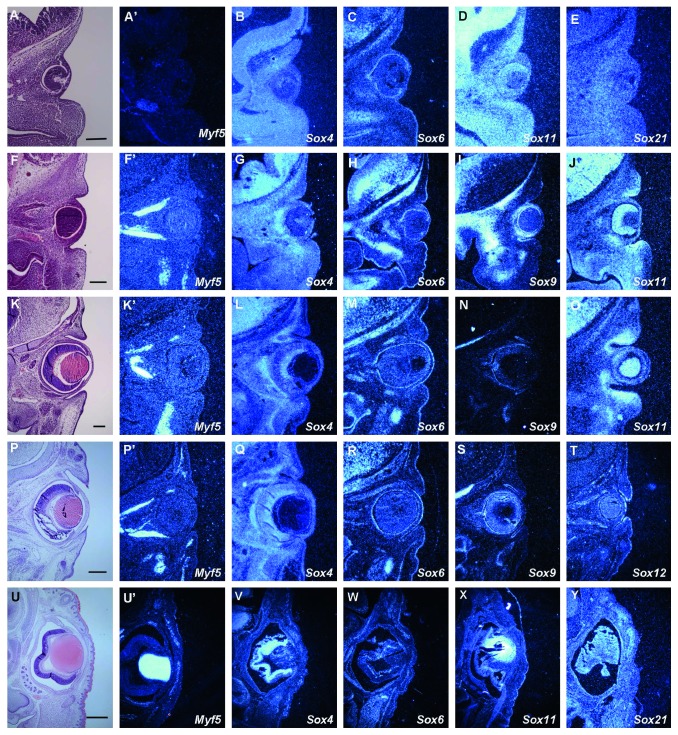
Expression of *Sox* genes in eyelid development. Frontal sections showing histology (a), (f), (k), (p), (u) and *in situ* hybridization ((a′)–(e), (f′)–(j), (k′)–(o), (p′)–(t), and (u′)–(y)) in wild type at E11 ((a)–(e)), E12 ((f)–(j)), E13 ((k)–(o)), E14 ((p)–(t)), and E18 ((u)–(y)). Scale bar: 150 *μ*m for (a)–(e), 125 *μ*m for (f)–(j), 125 *μ*m for (k)–(o), 300 *μ*m for (p)–(t), 700 *μ*m for (u)–(y).

Among the craniofacial organs, the eyelid shows some similarities with the tongue: Both are mobile organs and develop through epithelium, neural crest-derived mesenchyme, and mesoderm [2, 3, 19, 20]. In addition, it has been shown that the lack of Shh signaling pathway and microRNAs result in developmental defects of both the eyelid and the tongue [21–25]. These features suggest the possibility that tongue and eyelid development are under similar molecular mechanisms. To understand whether *Sox* genes are also involved in other craniofacial organs through similar roles as those in tongue development, we examined the expression of *Sox* genes in eyelid development. Murine eyelid initiates by groove formation at approximately E11, and the protrusion of the eyelid primordia occurs from E12 (Figures 6, 6, 5, and 6) [20]. Fusion of eyelid is observed at approximately E16, since the mammalian eyes require temporary fusion and re-opening between the upper and lower eyelids during their development and growth (Figure 6(u)). *Myf5* expression was observed in the eyelid primordia only at E14 and E18 (Figures 6, 6, 6, 6, and 6). At E11, *Sox4, 11,* and *21* showed ubiquitous expressions in the developing eyelid, whereas *Sox6* was only expressed in the eyelid epithelium (Figures 6–6). At E12 and E13, *Sox4* and *11* expressions were observed in both the epithelium and mesenchyme, whereas *Sox6* and *9* were only expressed in the epithelium (Figures 6–6 and 6–6). At E14, *Sox4* was ubiquitously expressed in the developing eyelid, with a slightly stronger expression in the muscles (Figure 6). *Sox6, 9,* and *12* were expressed in the epithelium of eyelid primordia, whereas *Sox11* expression was observed in both the epithelium and mesenchyme (Figures 6–6), data not shown). At E18, *Sox21* was ubiquitously expressed in the developing eyelid, with a slightly stronger expression in the muscles (Figure 6). Expressions of *Sox4* and *6* could be observed in the muscle of eyelid primordia, whereas *Sox11* was expressed in the epithelium (Figures 6–6). *Sox4, 6,* and *11* were also expressed in the extraocular muscle region. Expressions of *Sox1–3, 5, 8, 10, 13,* and *14* could not be detected in the developing eyelid (data not shown).

## 4. Discussion

**Figure 7:**
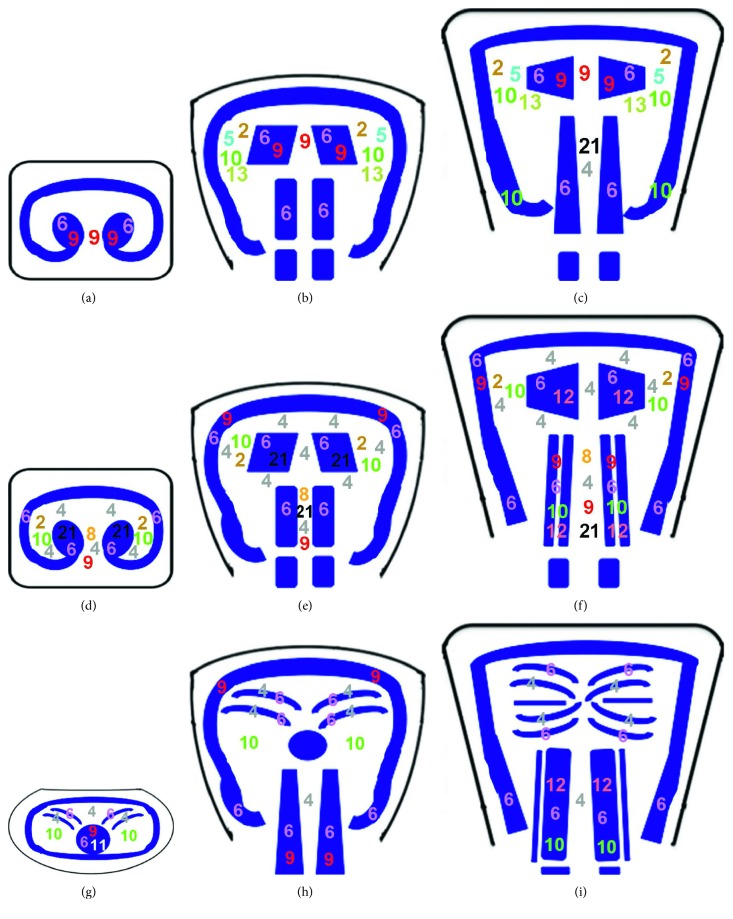
Summary of expression of *Sox* genes in tongue mesenchyme and muscle during development. Diagrammatic representation of developing tongue of the anterior (a), (d), (g); middle (b), (e), (h); and posterior region (c), (f), (i) at E12 (a–c), E13 (d–f), and E14 (g–i). The muscle is shown in blue. The numbers represent each *Sox* gene. *Sox* genes showing ubiquitous expression are excluded.

The mammalian tongue is composed of the epithelium, connective tissue, and striated muscle. Our comparative *in situ* hybridization analysis demonstrated a dynamic spatiotemporal expression pattern of *Sox* genes during murine tongue development. *Sox2* and *11* were expressed in the tongue epithelium during all five stages investigated in this study, whereas *Sox8* and *9* were expressed in the tongue epithelium only at E14 and E12, respectively. *Sox4, 6, 12,* and *21* showed expressions in the epithelium at several stages (Table 2). *Sox4* and *21* were expressed in the mesenchyme at all stages, whereas *Sox5, 6,* and *13* were only detected in the mesenchyme at E12 (*Sox5, 13*) and E18 (*Sox6*). *Sox2, 8, 9, 10,* and *11* showed detectable expressions in the mesenchyme at several stages (Table 2). Unlike the epithelium and mesenchyme, none of the *Sox* genes were expressed in the tongue muscle during all five stages. *Sox1* was expressed in the tongue muscle only at E18. *Sox6* and *10* were not detected in the muscle at E11 but were expressed during the other four developmental stages (E12–E18). Additionally, *Sox11* was not expressed in the muscle at E18 but was expressed during the other four developmental stages (E11–E14). *Sox4, 8, 9, 12,* and *21* showed detectable expressions in the muscle at several stages (Table 2). *Sox1* was only expressed in the muscle, whereas *Sox5* and *13* were expressed only in the mesenchyme. *Sox2* could not be detected in the muscle at any stage, whereas *Sox12* showed no expression in the mesenchyme at any stage. Although the expressions of *Sox2, 4, 10, 11,* and *21* were detected in the developing tongue during all five stages investigated in this study, their expression patterns differed among the stages. Our results also revealed a different expression pattern of *Sox* genes between the anterior, middle, and posterior region of the developing tongue (Figure 7). *Sox* genes thus showed a dynamic spatiotemporal expression pattern during murine tongue development.

Fungiform, circumvallate, and foliate papillae contain taste buds and are referred to as taste (or gustatory) papillae, whereas filiform papillae do not contain taste buds and are thus regarded nongustatory. Although *Sox2* has been shown to play a critical role in regulating the formation of gustatory papillae, its expression has also been detected in the nongustatory tongue epithelium [14]. Our results revealed that *Sox2, 4, 6, 8, 9, 10, 11, 12,* and *21* were expressed in the epithelium of the entire tongue. Although none of these genes showed a restricted expression pattern in the tongue papillae, it is possible that they are involved in regulating the formation of all papillae types.

Skeletal muscle fibers are classified as “slow-twitch fiber” (type I) and “fast-twitch fiber” (type II), which display marked differences in contraction strength, metabolic strategies, and susceptibility to fatigue. Slow-twitch fibers are rich in mitochondria, have increased contraction endurance with lesser strength potential, and use predominantly oxidative phosphorylation for energy production. Fast-twitch fibers contain comparatively less numbers of mitochondria and rely more heavily on anaerobic glycolysis for energy production, which allows considerable strength and contraction speed, but only for short anaerobic bursts of activity before the muscles fatigue. It has been reported that *Sox6* is involved in fast-twitch muscle fiber differentiation in the trunk region [9, 10]. It is known that rodent tongue is composed of only fast-twitch fiber, and we also found *Sox6* expression in the muscles of the developing tongue [26, 27]. These suggest that *Sox6* is likely to be involved in fast-twitch muscle fiber differentiation during tongue development. *Sox8, 9, 10, 12,* and *21* were observed in the tongue muscles where *Sox6* was expressed, suggesting the possibility that these *Sox* genes also regulate fast-twitch muscle fiber differentiation. On the other hand, unlike the mouse tongue, the human tongue shows high proportion of slow-twitch fibers, suggesting that the role of *Sox* genes differs between the human and mouse tongue [28].

The developmental origin of the masticatory muscles is the cardiopharyngeal mesoderm, while the tongue and other muscles, including the trunk and limb muscles, are derived from the somites [29]. Although the embryonic origins are the same in the tongue, limb, and trunk muscle, it has been shown that the program controlling tongue myogenesis, including the specification and migration of muscle progenitor cells, differs from those of the trunk and limb myogenesis [2, 3, 30]. Therefore, we could not exclude the possibility that the role of *Sox* genes expressing in developing tongue muscle differ from that in trunk and limb muscle development.

The tongue consists of many types of muscles, including vt, sil, and gg muscles [2, 30, 31]. Although our results indicated that many *Sox* genes were expressed in these tongue muscles, there was no specific *Sox* gene expressing in the particular muscles (Figure 7). *Sox* genes are therefore unlikely to play a critical role in tongue muscle specification. Different expression patterns between *Sox* genes in each tongue muscle type suggested that each muscle type is controlled by different molecular mechanisms during development.

Skeletal myogenesis consists of six phases: specification, migration, proliferation, determination, differentiation, and maturation [1, 2, 29–31]. It is unlikely that each phase of myogenesis proceeds at the same time in all types of tongue muscle since the timing of appearance differs between muscle types in the tongue [1, 2, 29–32]. It is possible that the different expression patterns of *Sox* genes in tongue muscles could be correlated with the onset of these stages, although the molecular mechanisms regulating the initiation of each phase during tongue development remain unclear. It has been shown that *Sox7, 15, 17* and *18* are involved in the function of satellite cells after birth, although these are not exerted at embryonic stages [11–13]. Therefore, a punctate expression pattern of *Sox7, 17,* and *18* observed in the embryonic tongue are likely to represent vascularization of the tissue. Furthermore, these also indicated that function of *Sox* genes differ between embryonic and postnatal stages in tongue formation.

Previous studies have reported that neural crest-derived cells give rise to tissues surrounding the skeletal muscles, including the perimysium, epimysium, endomysium, and tendon, during tongue development [33, 34]. *Sox* genes have been shown to regulate the functions of neural crest-derived cells including their differentiation, collagen synthesis, and melanocyte formation [35, 36, 37]. In our study, *Sox9* displayed a restricted expression pattern in the lingual septum, which is composed of neural crest-derived cells [2, 3]. It has been reported that *Scleraxis* is expressed in a similar pattern to that of *Sox9* during tongue development [2, 32]. Interestingly, *Sox9* shows coordinated expression with *Scleraxis* during digital tendon development [38]. Therefore, it is possible that *Sox9/Scleraxis-*expressing cells are involved in the formation of tongue muscle tendons.

The origin of the tongue is known to be hybrid, neural crest-derived cells, the mesoderm, and endoderm. It has been shown that there is an interaction between neural crest-derived and myogenic cells during tongue development [2, 3]. We detected the expressions of *Sox1, 4, 6, 8, 9, 10, 11, 12, 13,* and *21* in the muscles of the developing tongue and of *Sox2, 4, 5, 6, 8, 9, 10, 11,* and *21* in the mesenchyme. It is possible that these genes are involved in the interaction between neural crest-derived and myogenic cells during tongue development. In fact, some *Sox* genes have been proven to be involved in the interaction between the epithelium and mesenchyme in digestive tract development [39, 40].

Members of the *Sox* gene family play multiple roles during development [5, 6]. The dynamic spatiotemporal expression pattern of *Sox* genes discovered in this study indicates their critical role during murine tongue development. We also examined the expression of *Sox* genes in eyelid development (Table 2). Both organs consist of connective tissue, muscle, and epithelium, and there is an interaction between these tissues during their development [2, 3, 19, 20]. In addition, both tongue and eyelid are mobile organs. Although there are thus numerous similarities between the eyelid and tongue, our results showed that the expression patterns and timing of *Sox* genes significantly differed between tongue and eyelid development. *Sox* genes are thus likely to be involved in organogenesis through different functions in each organ.

## 5. Conclusion

*Sox* genes show a dynamic spatiotemporal expression pattern during murine tongue development.

## Figures and Tables

**Table 1:** Probe information.

	Size of fragment of cDNA	Exon
*Sox1*	255 bp	1
*Sox2*	700 bp	1
*Sox3*	900 bp	1
*Sox4*	2.9 kb	1
*Sox5*	1.5 kb	1–11
*Sox6*	5.0 kb	2–16
*Sox7*	867 bp	2
*Sox8*	780 bp	1–3
*Sox9*	510 bp	3
*Sox10*	2.5 kb	1–4
*Sox11*	3 kb	1
*Sox12*	900 bp	1
*Sox13*	3.3 kb	2–11
*Sox14*	1.9 kb	1
*Sox17*	960 bp	4,5
*Sox18*	856 bp	2
*Sox21*	714 bp	1

**Table 2:** Summary of expression of *Sox* genes in developing tongue and eyelid.

Tongue epithelium												
E11	—	*Sox2*	*Sox4* ^*∗*^	—	—	—	—	—	*Sox11* ^*∗*^	—	—	*Sox21* ^*∗*^
E12	—	*Sox2*	—	—	*Sox6*	—	*Sox9*	—	*Sox11* ^*∗*^	—	—	—
E13	—	*Sox2*	—	—	*Sox6*	—	—	—	*Sox11* ^*∗*^	*Sox12*	—	—
E14	—	*Sox2*	*Sox4*	—	*Sox6*	*Sox8* ^*∗*^	—	—	*Sox11*	*Sox12*	—	*Sox21* ^*∗*^
E18	—	*Sox2*	*Sox4*	—	*Sox6* ^*∗*^	—	—	*Sox10*	*Sox11*	—	—	*Sox21* ^*∗*^

*Tongue mesenchyme*												
E11	—	*Sox2*	*Sox4* ^*∗*^	—	—	—	—	*Sox10*	*Sox11* ^*∗*^	—	—	*Sox21* ^*∗*^
E12	—	*Sox2*	*Sox4*	*Sox5*	—	—	*Sox9*	*Sox10*	*Sox11* ^*∗*^	—	*Sox13*	*Sox21*
E13	—	*Sox2*	*Sox4*	—	—	*Sox8*	*Sox9*	*Sox10*	*Sox11* ^*∗*^	—	—	*Sox21*
E14	—	—	*Sox4*	—	—	*Sox8* ^*∗*^	—	*Sox10*	—	—	—	*Sox2*1^*∗*^
E18	—	—	*Sox4*	—	*Sox6* ^*∗*^	—	—	—	—	—	—	*Sox21* ^*∗*^

*Tongue muscle*												
E11	—	—	*Sox4* ^*∗*^	—	—	—	—	—	*Sox11* ^*∗*^	—	—	*Sox21* ^*∗*^
E12	—	—	—	—	*Sox6*	—	*Sox9*	*Sox10*	*Sox11* ^*∗*^	—	—	—
E13	—	—	—	—	*Sox6*	*Sox8*	*Sox9*	*Sox10*	*Sox11* ^*∗*^	*Sox12*	—	*Sox21*
E14	—	—	—	—	*Sox6*	*Sox8* ^*∗*^	*Sox9*	*Sox10*	*Sox11*	*Sox12*	—	*Sox21* ^*∗*^
E18	*Sox*1	—	*Sox4*	—	*Sox6* ^*∗*^	*Sox8*	—	*Sox10*	—	*Sox12*	—	*Sox21* ^*∗*^

*Eyelid mesenchyme*												
E11	—	—	*Sox4* ^*∗*^	—	—	—	—	—	*Sox11* ^*∗*^	—	—	*Sox21* ^*∗*^
E12	—	—	*Sox4*	—	—	—	—	—	*Sox11*	—	—	—
E13	—	—	*Sox4*	—	—	—	—	—	*Sox11*	—	—	—
E14	—	—	*Sox4* ^*∗*^	—	—	—	—	—	*Sox11*	—	—	—
E18	—	—	—	—	—	—	—	—	—	—	—	*Sox21* ^*∗*^

*Eyelid muscle*												
E14	—	—	*Sox4* ^*∗*^	—	—	—	—	—	—	—	—	—
E18	—	—	*Sox4*	—	*Sox6*	—	—	—	—	—	—	*Sox21*

^*∗*^Ubiquitous expression.

## Data Availability

The data used to support the findings of this study are included within the article.
